# Spark Plasma Sintering of Fine-Grained WC-Co Composites

**DOI:** 10.3390/ma16247526

**Published:** 2023-12-06

**Authors:** Joanna Wachowicz, Tomasz Dembiczak, Joanna Fik, Zbigniew Bałaga, Robert Kruzel, Nataša Náprstková, Sylvia Kuśmierczak

**Affiliations:** 1 Department of Mechanical Processing of Wood, Institute of Wood Sciences and Furniture, Warsaw University of Life Sciences, Nowoursynowska Street, 166, 02-787 Warsaw, Poland; 2 Faculty of Science and Technology, Jan Dlugosz University in Czestochowa, Armii Krajowej Street 13/15, 42-200 Czestochowa, Poland; j.fik@ujd.edu.pl; 3 Faculty of Production Engineering and Materials Technology, Czestochowa University of Technology, Armii Krajowej Street, 19, 42-201 Czestochowa, Poland; zbigniew.balaga@pcz.pl; 4 Faculty of Civil Engineering, Czestochowa University of Technology, Akademicka Street 3, 42-201 Czestochowa, Poland; robert.kruzel@pcz.pl; 5 Faculty of Mechanical Engineering, Jan Evangelista Purkyně University in Ústí nad Labem, Pasteurova Street 1, 400 96 Ústí nad Labem, Czech Republic; natasa.naprstkova@ujep.cz (N.N.); sylvia.kusmierczak@ujep.cz (S.K.)

**Keywords:** spark plasma sintering, cemented carbides, WC-Co, abrasive wear resistance

## Abstract

Cemented carbides WC-Co are one of the basic tool materials. They constitute over half of the currently used tools intended for machining. The main advantages of WC-Co cemented carbides are high hardness and abrasion resistance. The properties of WC-Co sinters depend mainly on the content of the binding phase, the sintering method and the grain size of the powder from which the sinters were made. The aim of this study was to produce fine-grained WC-Co composites using SPS (spark plasma sintering) technology, as well as examine the effect of the applied technology on the basic properties of WC-Co sinters: microstructure, hardness, phase composition, compaction degree and tribological properties. In the processes carried out, no additives affecting the reduction in grain growth were used. Sintering was conducted at a temperature of 1200 °C with a holding time of 10 min. The process occurred under a load of 100 MPa. Finally, the samples were cooled in a vacuum of 10^−6^ mbar. We measured the hardness using a Vickers hardness tester. We took hardness measurements along the diameter of the sintered samples. In order to ascertain the fracture toughness (KIC), we measured the radial crack length around the Vickers indentation and applied Shetty’s formula. The tribological tests were carried out with a tribotester using the T-01 ball-on-disc method. The obtained data enabled the characterization of the wear process of the tested materials.

## 1. Introduction

Cemented carbides are one of the most important tool materials. They consist of a hard tungsten carbide (WC) phase and a cobalt (Co) binder. WC-Co composites offer a unique combination of properties, such as high hardness and resistance to brittle fracture. Sintered tungsten carbide, which comprises large proportions of WC particles in a cobalt metal skeleton, is classified as a cermet composite material. It is well known that the hardness of WC-Co composites decreases with increasing grain size and that fracture toughness decreases with hardness. Therefore, the harder the material, the more brittle it is. It can also be concluded that hardness is dependent on the average diameter of the tungsten carbide crystals. The material with the smallest grain size is the hardest [1]. However, the link between hardness and fracture toughness may not follow a linear pattern when the grain sizes are extremely small [2,3,4,5,6].

The properties that characterize WC-Co composites stipulate their employment predominantly as cutting tools. Recently, there has been an increasing focus on ultra-fine and nanocrystalline cemented carbides [7]. Sintered tungsten carbide bearing a nanocrystalline structure provides a considerable improvement to the mechanical properties of these materials. Using nanopowders to enhance the properties of WC-Co composite tools could significantly increase their lifespan and durability. Nanopowder-based materials exhibit much higher hardness than those made with conventional powders. However, literature concerning the fracture toughness of WC-Co nanocomposites is not uniform, and thus, it remains unclear whether nanopowder materials confer an advantage in terms of fracture toughness. It should be acknowledged that the potential of a fully consolidated nanometric WC-Co composite has yet to be fully investigated [8,9,10,11,12,13,14].

The characteristics of WC-Co composites are affected by various factors, including the sintering procedure, powder composition, and inhibitor selection and concentration. Advanced consolidation techniques, such as electric-field-assisted sintering, microwave sintering and spark plasma sintering, were devised to produce ultrafine or nanometric structures of these materials [15,16,17,18].

The production of ultrafine composites faces technical obstacles due to the high reactivity and rapid growth of ultrafine/nanopowders during sintering. The growth of WC grains can be limited by transition metal carbides, resulting in a uniform distribution of the Co phase and enhanced mechanical properties of the sintered carbide. Additionally, certain rare earth elements can function as additives that partially obstruct WC grain growth during the process of sintering. They simultaneously promote the purification of grain boundaries and enhance interfacial bonding, which subsequently leads to an enhancement of mechanical properties in the sintered carbide. The ultimate purpose of these growth inhibitors is to suppress the dissolution–re-precipitation or Ostwald maturation process. Typically, up to 2% wt. of inhibitor addition is utilized [7,19].

Spark plasma sintering (SPS) is a pressurized method that uses DC pulses to efficiently create high-temperature discharges between sintered material particles. This results in a fast sintering process. The method’s self-heating mechanism for the sintering powder allows for heat to be released exactly where it is required for sintering, reducing the entire process time. During the passage of an electric current, the material’s temperature rises to a high level, and it rapidly drops to the sintering temperature when the current disappears. This energy delivery method makes the process thermally very efficient. The Joule heat and spark discharges ignited in the pores between individual powder particles warm the powder. The advantages of this method over conventional sintering methods, like HP (hot press) or HIP (hot isostatic press), include easy usage, accurate control over sintering energy, fast sintering, rapid heating and cooling of the sintered material, reduced energy consumption, low costs, high repeatability, safety and reliability [20].

Studies indicate that the nanoscale properties of carbides are lost when sintered using conventional methods due to rapid grain growth during the consolidation process. Although ultra-fine-grained commercial WC-Co composites are now available, it is still a significant technological challenge to control grain growth during sintering and produce hard nanocrystalline materials. Manufacturing challenges include economic considerations, as well as the powder properties and processability [2,21,22,23,24,25,26,27,28,29].

Conventional sintering processes for WC-Co composites necessitate the employment of grain growth inhibitors (such as VC and Cr_3_C_2_) to prevent WC grain growth while sintering [30,31,32]. However, it is important to note that the amount of inhibitor should not exceed 2 wt%. To attain uniform mixing, a lengthy ball milling process is necessary, but this may lead to a greater number of defects, resulting in anomalous WC grain growth during subsequent compaction procedures. It is challenging to achieve a WC-Co composite with a precise distribution of WC grains and an ultra-fine structure [33].

In this study, spark plasma sintering was used to produce WC-Co composites. The effects of the grain size of the WC starting powder on the resulting microstructure and chemical and mechanical properties of the materials were examined. The composites were characterized by means of X-ray diffraction and scanning electron microscopy. Furthermore, investigations into the hardness, K_IC_ fracture toughness factor and tribological properties were conducted.

## 2. Experimental Procedure

High-purity, commercially available blends of WC and Co powders were utilized in this research. An analysis of the chemical composition of the powders to be sintered, as disclosed by the supplier, is shown in Table 1 and Table 2. The powders had average WC grain sizes of 0.8 µm, 0.4 µm and 0.1 µm. Sinters containing 4 wt% cobalt were employed for WC grain sizes of 0.8 µm and 0.4 µm, while 5 wt% cobalt was used for sinters with a WC grain size of 0.1 µm. Table 3 outlines the different types of composites tested during the study.

Powder mixtures were placed in a graphite die for the SPS (spark plasma sintering). The mixture was heated using electric current pulses flowing through the graphite elements and the powder. The SPS method reduces the sintering temperature and time compared with traditional methods, resulting in reduced grain growth.

**Figure 1:**
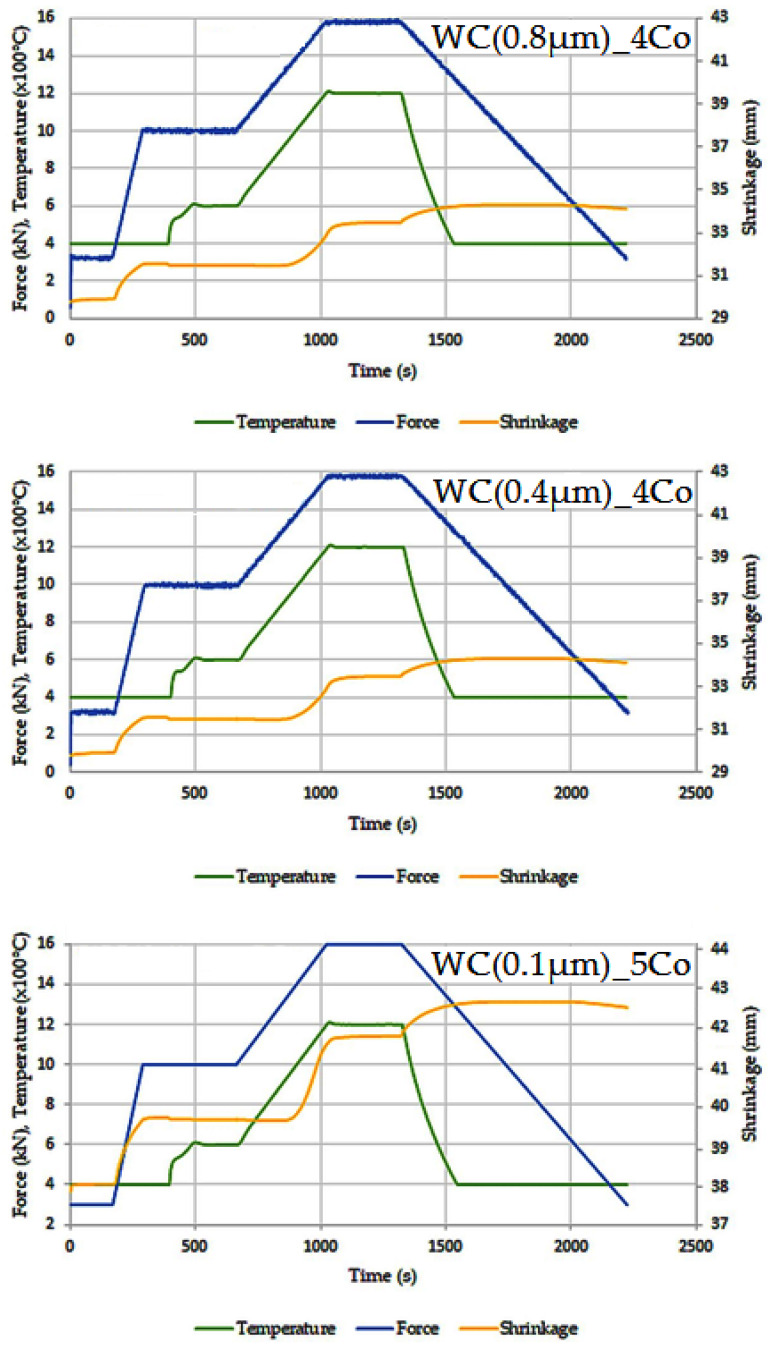
Temperature, force and shrinkage cycles during SPS process of WC-Co composites.

The WC-Co composites were consolidated through the sintering process using the SPS spark plasma sintering machine from FCT Systeme Gmbh (Effelder-Rauenstein, Germany). The consolidation process was divided into two stages. In the first stage, degassing was conducted at a temperature of 600 °C, followed by the second stage at 1200 °C for a duration of 10 min. The pressing pressure was raised from 30 MPa to 50 MPa, and the whole process was executed under vacuum conditions. Figure 1 displays the graphical representation of the consolidation processes.

Microstructure images of the fracture test samples were captured using a JEOL 6610LV scanning microscope (JEOL Ltd., Tokyo, Japan) equipped with a LaB6 cathode. The hardness was measured using a Vickers hardness tester under a constant load of 30 kg. For the fracture toughness (K_IC_), the radial crack length around the Vickers indentation was measured and the Shetty formula was applied [34]:

(1) $$K_{IC}=A\sqrt{H}(\frac{P}{\sum L})$$

where *H* is the hardness (N/mm^2^), *P* is the applied load (N), Σ*L* is the sum of the crack lengths (mm), *A* is a constant with a value of 0.0028 and *K_IC_* is given in MPa m^1/2^. For HV30 values expressed in kgf/mm^2^, the Palmqvist fracture toughness can be calculated using

(2) $$K_{IC}=0.15(\frac{{HV}_{30}}{\sum L})$$

X-ray diffraction was employed to analyze the WC-Co composites, utilizing a SEIFERT XRD 3003 T-T instrument (SPECTRO Analytical Instruments, Kleve, Germany) equipped with a cobalt anode lamp.

The tribological tests were conducted using a T-01 tribological tester and the ball-on-disc method with a 10 mm diameter Al_2_O_3_ ball. The tests were performed under dry friction conditions with a contact force of 55 N, a total friction path of 3000 m, a rotational speed of 500 rpm and a radius of 3 mm. The total friction path was used to determine the kinetics of abrasive wear through fifteen cycles with a distance of 200 m each. After each cycle, the samples were degreased with ethanol using an ultrasonic cleaner, equalized and weighed with an OHAUS PX125D balance (Ohaus, Parsippany, NJ, USA). The wear rate was calculated based on the weight loss (ΔG) of the samples after the entire abrasion cycle. The friction force was recorded during the measurements to determine the coefficient of friction. The samples’ surfaces were observed and the surface roughness parameters were evaluated using a Keyence VHX-7000 microscope (Keyence, Osaka, Japan) before and after the 5th, 10th and 15th cycles. The parameters evaluated were Ra—the arithmetic mean roughness value, Rt—the total height of the roughness profile and Rz—the mean depth of roughness, with a 3D image of the surface also taken. The obtained data helped in characterizing the wear process of the materials tested [35].

## 3. Results and Discussion

**Figure 2:**
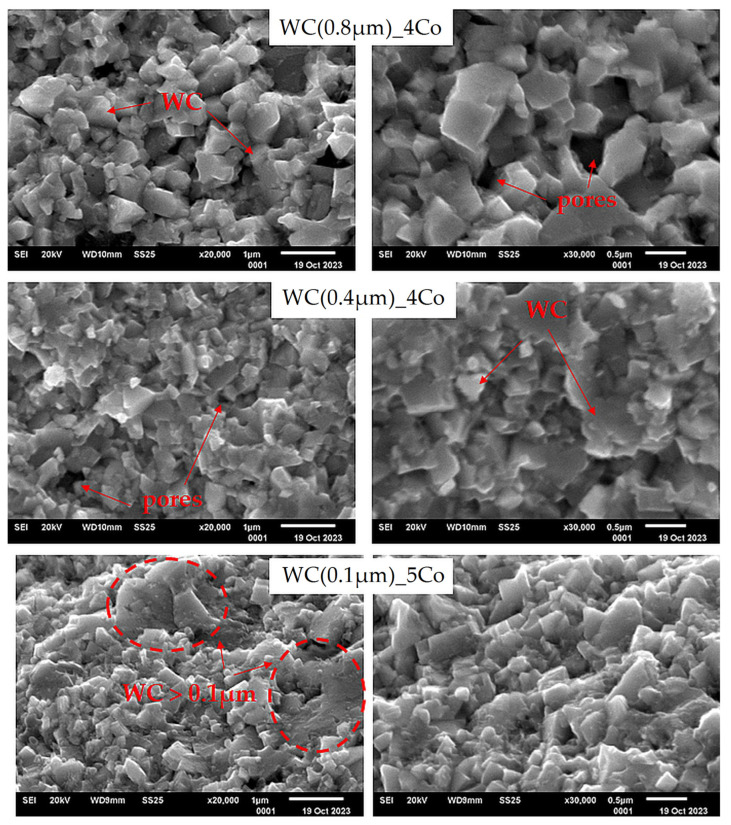
SEM images of the fracture surface of WC-Co composites.

Figure 2 shows the microstructure of the fractures of the obtained composites with different WC grain sizes. The observations show that in all the obtained sintered composites, the WC grains were well-shaped and had sharp edges. A slight porosity could be observed in all samples. The presence of pores indicates an insufficient distribution of plastic cobalt in the WC framework. In the images of the composites obtained from the powder with the finest WC gradation, areas where the WC grains grew can be observed.

Table 4 shows the results of the hardness measurements for the WC-Co composites tested. For the submicron composites (for WC grain sizes of 0.4 and 0.8 µm), the hardness decreased with increasing grain size according to the Hall–Petch relationship. The tests showed the highest HV30 hardness of 2224 ± 19 for composites with a WC grain size of 0.4 µm. For composites with a larger WC grain size of 0.8 µm, the average HV30 hardness was 2048 ± 34. The presence of post-sintered regions with a larger WC grain size in the WC (0.1 µm)_5Co sinters resulted in a decrease in hardness compared with the WC (0.4 µm)_4Co composites. It should also be noted that this group of sinters had a slightly higher cobalt content of 5% by weight.

Both hardness and fracture toughness are crucial mechanical properties of hard composites for cutting blades. It was observed that with the same cobalt content, both hardness and fracture toughness increased as the tungsten carbide (WC) grain decreased.

**Figure 3:**
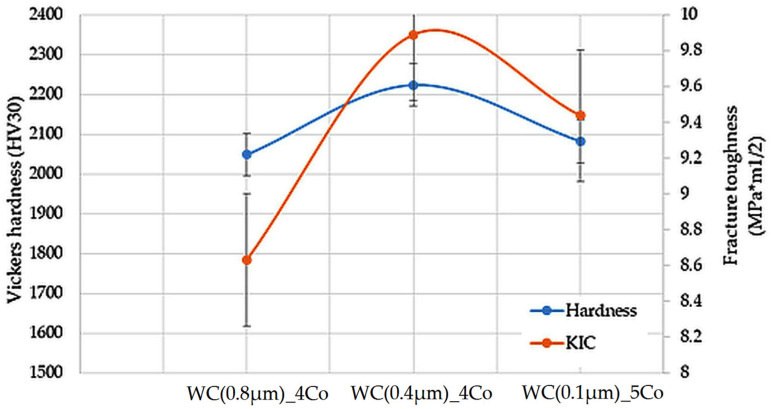
Vickers hardness and fracture toughness for the tested WC-Co composites.

The fracture toughness K_IC_ value for the WC(0.4µm)_4Co sinters was 9.89 MPa m^1/2^. However, in composites produced from the finest WC(0.1)µm_5Co powders, although there was a slightly higher cobalt content, the K_IC_ was lower at 9.47 MPa m^1/2^ (Figure 3). This may have been due to the partial growth of the WC grains in these samples, as shown in Figure 2. Studies in the literature point out that the presence of uniform WC grain sizes results in the best balance between hardness and fracture toughness. The presence of larger tungsten carbide grain sizes significantly affects the deflection of crack propagation [36,37].

**Figure 4:**
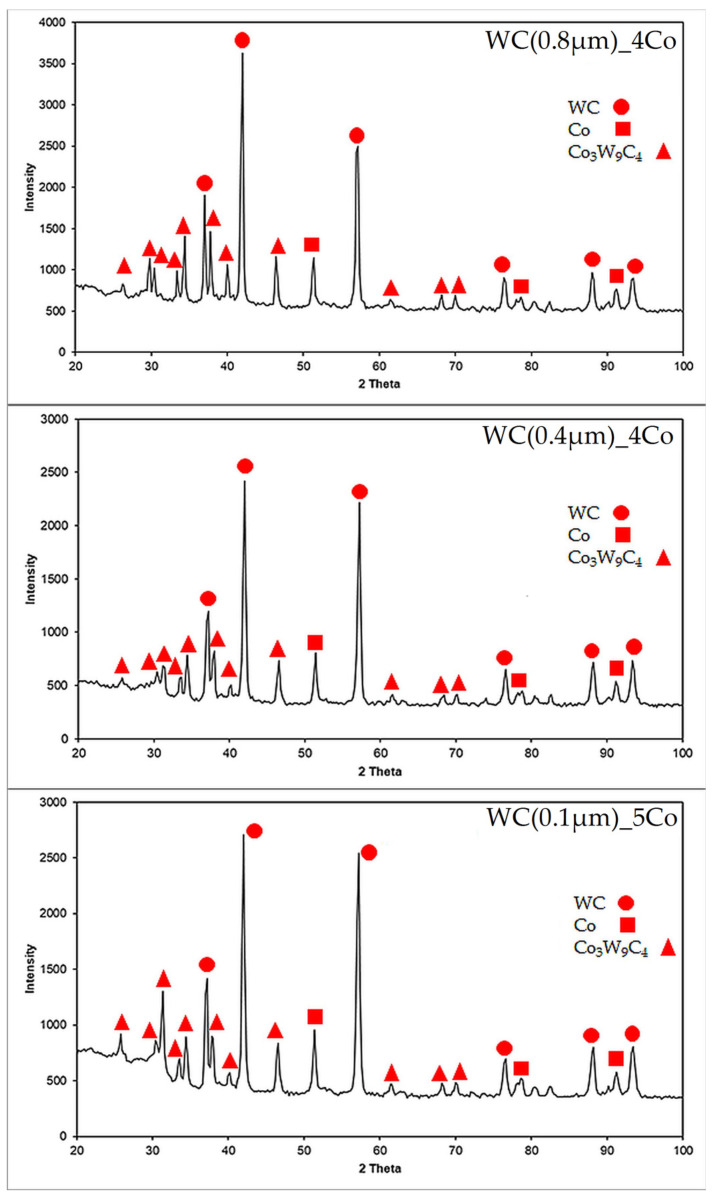
XRD patterns with a selected 2θ range of 20°–100° for WC-Co sinters (Co: 96-901-2885; WC: 96-210-2245; Co_3_W_9_C_4_: 96-152-8858).

The investigation of the phase composition in all the examined sinter variations revealed the presence of several phases, namely, WC, FCC Co and Co_3_W_9_C_4_, after sintering the micro-powder materials (Figure 4). A comparable phase composition was achieved when the same powders were sintered using a U-Fast device [34]. Regrettably, the presence of a ternary η Co_3_W_9_C_4_ phase was detected in all variants of the WC-Co composite investigated. The quantity of this phase may adversely affect the mechanical characteristics of the examined materials. Previous research demonstrated that the incorporation of suitable free carbon into the composite powders can hinder the formation of the η phase during the dissolution and precipitation of WC particles in the sintering process of the liquid phase. This, in turn, enhances the microstructure and mechanical properties of cemented carbides [36].

**Figure 5:**
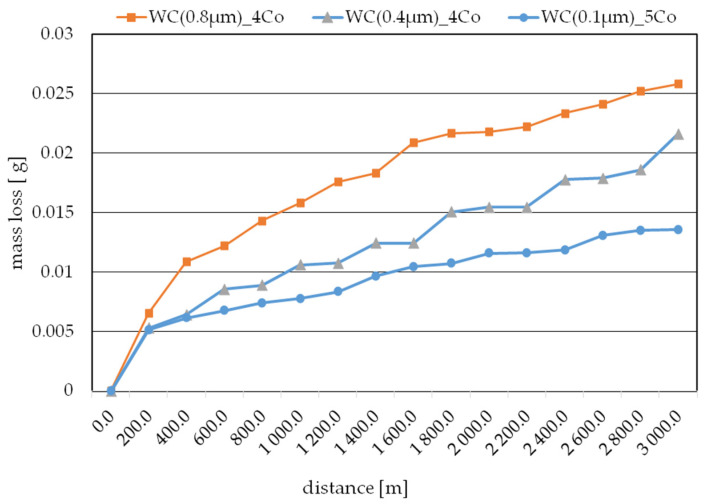
Change in weight of samples during successive friction cycles.

To establish the wear kinetics, Figure 5 illustrates the change in weight loss for the WC-Co composites subjected to successive friction cycles. The sample with the largest WC grain size, namely, WC(0.8µm)_4Co, experienced the most intensive wear. Thereafter, the WC(0.4µm)_4Co sample was used to determine the average wear rate, while the WC(0.1µm)_5Co composite exhibited the lowest weight loss.

**Figure 6:**
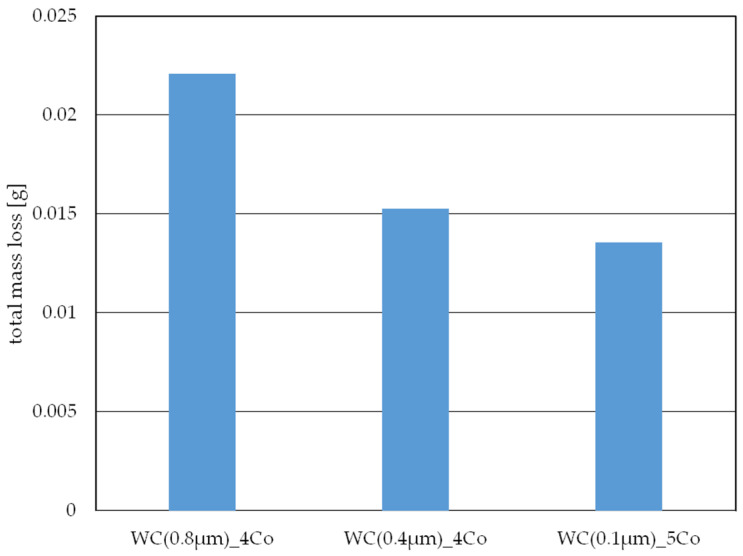
Total weight loss ΔG of a ball.

As the literature [38] shows, the degree of wear of a designed friction node is significantly influenced by many factors, including the components used in it. Durability depends on both the test material used and the choice of counter sample. The weighing method used to assess the degree of wear allowed the degree of wear of the counter sample, namely, an Al_2_O_3_ ball, to be determined. Figure 6 shows the total weight loss of the counter samples for each of the material variants tested. Analysis of the results obtained showed that in each of the cases tested, the weight loss of the bead was proportional to the loss of the materials tested.

**Figure 7:**
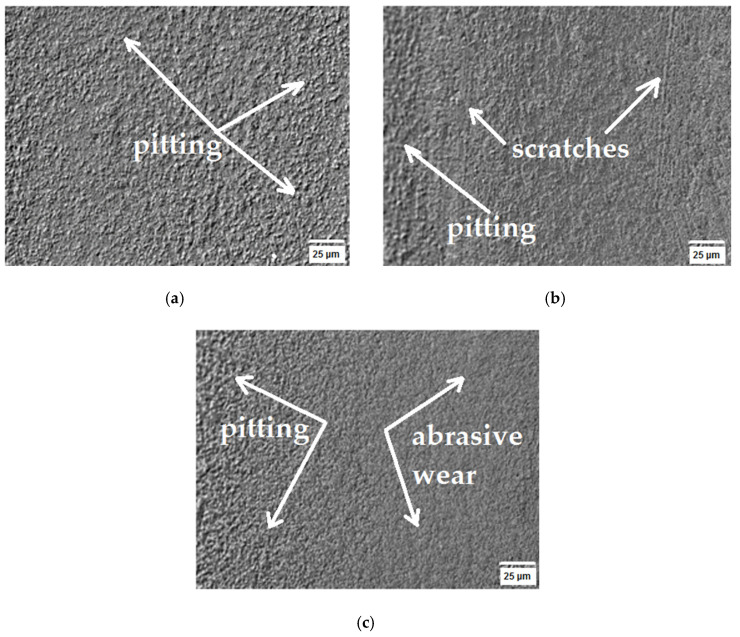
Topography of WC(0.8μm)_4Co sample found using SEM: (**a**) after 5th cycle, (**b**) after 10th cycle and (**c**) after 15th cycle.

**Figure 8:**
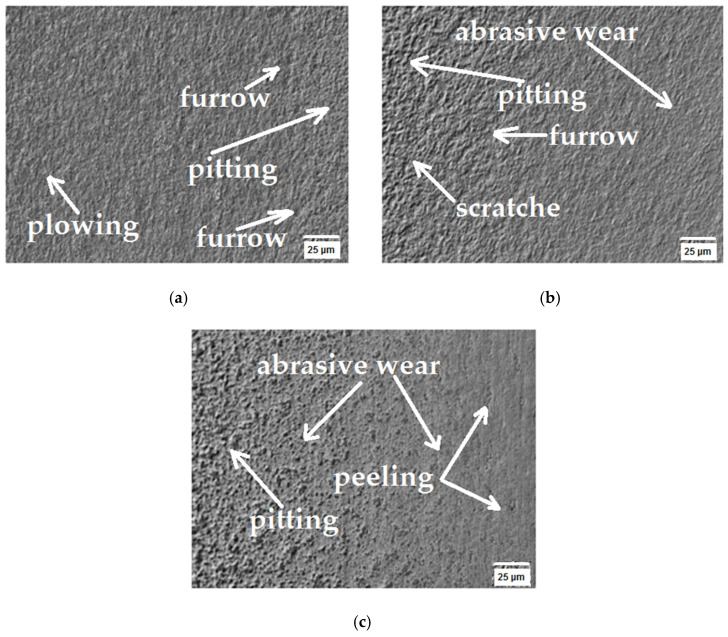
Topography of WC(0.4μm)_4Co sample found using SEM: (**a**) after 5th cycle, (**b**) after 10th cycle and (**c**) after 15th cycle.

**Figure 9:**
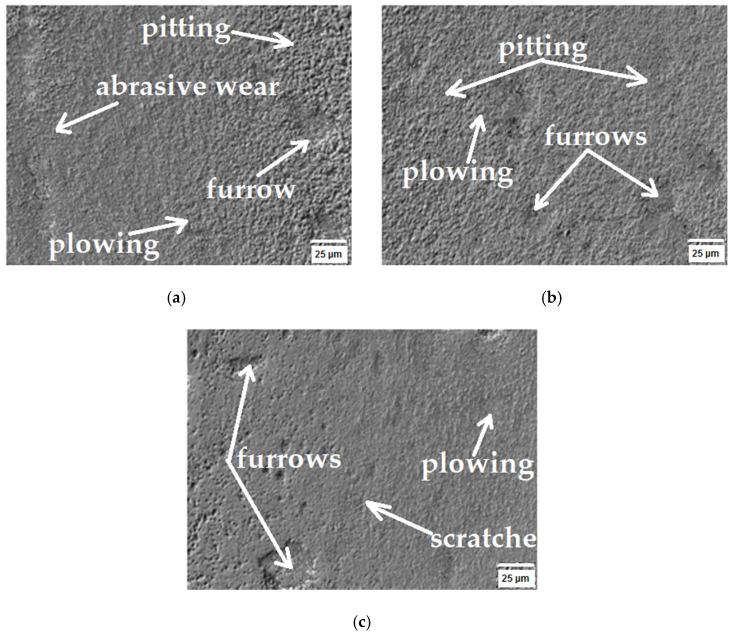
Topography of WC(0.1μm)_5Co sample found using SEM: (**a**) after 5th cycle, (**b**) after 10th cycle and (**c**) after 15th cycle.

To ascertain the tribological wear mechanism of the tested sinters, surface observations of the rubbing tracks were performed using a Keyence VHX-7000 microscope after 5, 10 and 15 cycles. The cooperation area surface texture is presented in Figure 7, Figure 8 and Figure 9. The obtained SEM images enabled identifying the wear nature, as they demonstrate the mechanical wear impact on the material surface in the friction node. Figure 7, Figure 8 and Figure 9 show the areas where surface changes occurred during successive cycles. The observations led to the conclusion that abrasion was the primary form of wear. The increase in wear was attributed to the detachment of material fragments that appeared in the contact area of the surface. The wear products took on the form of fragments detached from the surface, leading to visible losses for the WC(0.1μm)_5Co composite, as illustrated in Figure 9.

The microstructure image analysis was corroborated by reviewing the fundamental roughness parameters, as illustrated in Table 5.

The results of the measurements for the Ra and Rz parameters suggest that the surface of the WC(0.4μm)_4Co sample underwent increased smoothness after the initial five cycles, reflecting decreasing roughness parameters. Nonetheless, after the 10th cycle, Ra and Rz slightly increased, eventually reaching its maximum value after the 15th cycle. Providing a stark contrast, the WC(0.1μm)_5Co composite was the sole unit within the study that exhibited consistently stable roughness parameters, gradually decreasing as the number of cycles increased. After the 15th cycle, the Ra parameter was observed to be 30% lower than the initial value. The surface roughness parameters of the WC(0.8μm)_4Co material markedly increased in subsequent test cycles. Following the 15th cycle, parameters Ra and Rz were approximately fourfold higher than their initial values. The resultant surface image and total mass loss corresponded with these findings. 

**Figure 10:**
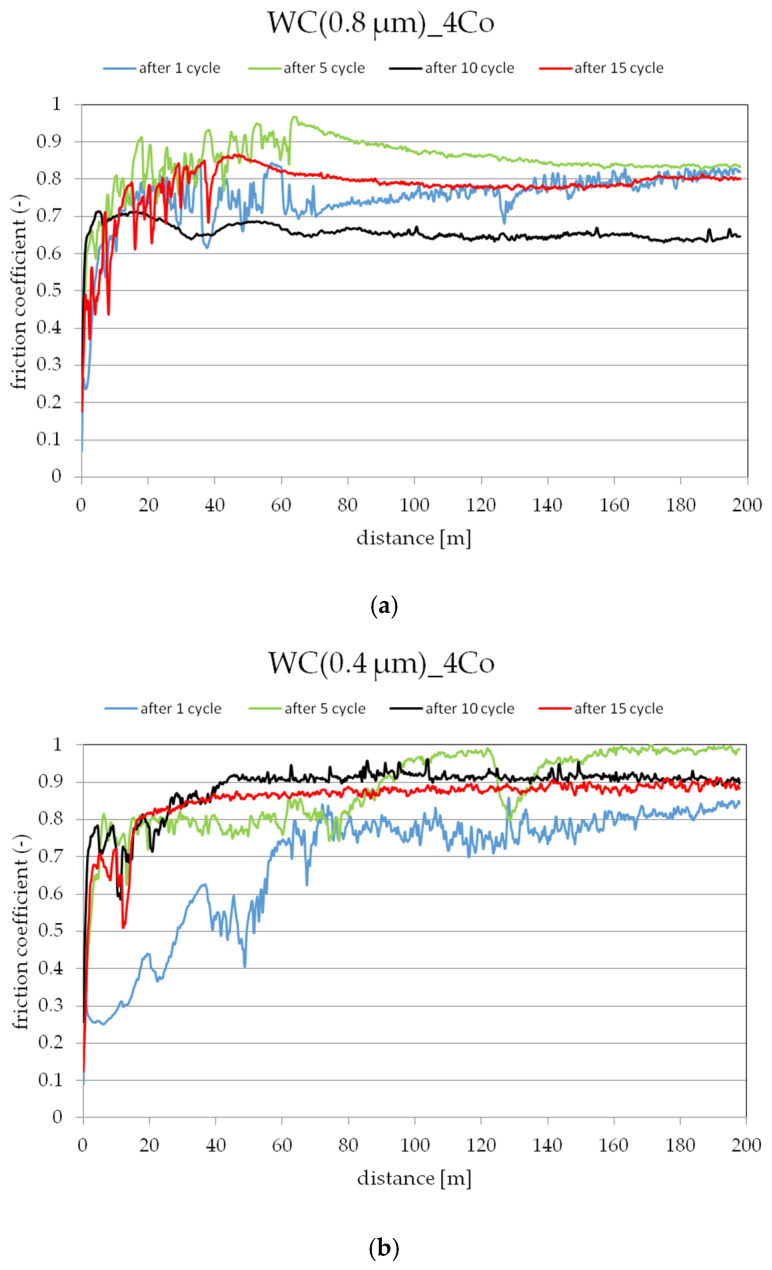

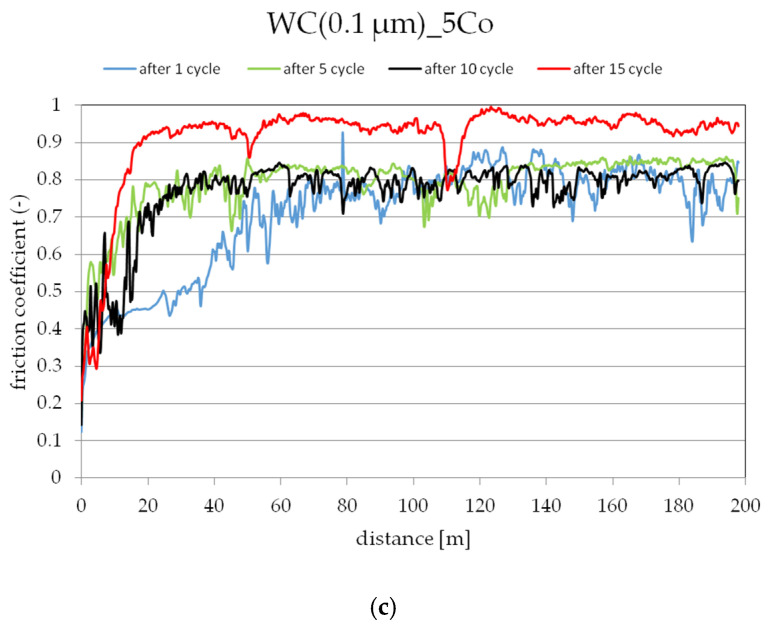
Coefficient of friction as a function of path length for sintering: (**a**) WC(0.8μm)_4Co, (**b**) WC(0.4μm)_4Co and (**c**) WC(0.1μm)_5Co.

Figure 10 displays the progression of friction coefficient characteristics throughout the different stages of tribological wear.

A sinter characterized by an initial average tungsten carbide (WC) particle size of 0.8 μm and a cobalt (Co) matrix content of 4% (Figure 10a) was characterized by a variable coefficient of friction as a function of distance traveled. After the first cycle, it was observed that lapping of the sinter surface took place. The unsteady jumps in the coefficient of friction during the first phase of abrasive wear over a distance of 70 m were caused by the exposure of hard WC particles on the sinter surface, resulting in spikes in frictional forces during the test. After a distance of 70 m was exceeded, it was most likely the case that the hard WC particles were dotare, resulting in a stabilization of the friction coefficient course. After the fifth test cycle, the observed cause of the abrasive wear was the resulting pitting visible on the sintered surface (Figure 7a). After the tenth test cycle, scratches and pitting were observed on the sinter surface (Figure 7b). After the tenth test cycle, a stabilized friction coefficient was observed in the range of 0.6 to 0.7. A stabilized course of sinter weight loss was also observed between the eighth and eleventh test cycles (Figure 5). 

After the fifteenth test cycle, an unstable increase in friction force was observed at the initial stage of the test, which was most likely due to the fact that the soft Co phase was wiped out faster and the hard WC particle phase was revealed on the sinter surface. The average coefficient of friction after a distance of 2967.3 m was 0.788. When analyzing the mass loss of the sinter WC(0.8μm)_0.4Co, it was predicted that the material had not been fully consumed, as evidenced by a further increase in the mass loss of the sinter, as shown in Figure 1. Based on the roughness analysis, an increase in the parameters, i.e., Ra and Rz, was observed for each test cycle (Table 5). Based on the observation of the images shown in Figure 7a–c, it was concluded that the only wear phenomenon observed was abrasive wear, whose cause was revealed to be scratches and pitting on the sinter surface [39,40].

Characteristics of the friction coefficient course for a sinter with an average initial WC particle size of 0.4 μm and a Co content of 4% are shown in Figure 10b.

The course of the friction coefficient between the first and fifth test cycles was unstable; there was an increase in the frictional forces, which may have been due to the fact that hard WC particles were revealed on the surface of the sinter. It was observed that the curvature character of the weight loss between the first and fifth test cycles was smoother than that of the WC(0.8μm)_0.4Co sinter (Figure 5), which also resulted in a smaller weight loss of the WC(0.4μm)_0.4Co sinter. Based on this observation, it was noted that the roughness parameters (Ra, Rz) decreased from the initial state, which may mean that there was a smoothing in the surface area of the sinter wipe tracks.

A stabilized state of the friction coefficient was observed between the tenth and fifteenth test cycles. This was due to the fact that no hard WC particles were revealed on the surface of the sinter, which may indicate the homogeneity of the structure. 

The stabilized average coefficient of friction after a distance of 2967.3 m was 0.882. When analyzing the mass loss of the WC(0.4μm)_0.4Co sinter, it was found that the sinter did not fully wear, as evidenced by the kinetics of the mass loss curve (Figure 5). Based on the analysis of the roughness parameters, a slight increase from the initial condition was observed. Based on the observation of the sinter surface (Figure 8a–c), it was found that the wear phenomenon was classic abrasive wear formed by scratches, pitting, plowing and furrowing [41,42].

The characteristics of the friction coefficient for a sinter with an average initial WC particle size of 0.1 μm and a Co content of 5% are shown in Figure 10c.

The friction coefficient characteristics between the first and fifteenth test cycles were similar to each other. The increases in frictional forces occurred due to the exposure of hard WC particles on the sinter surface. On the basis of the analysis of sinter weight loss, a smooth curve was observed, which indicates a uniform weight loss without sharp increases. The observed wear-associated phenomenon was the loss caused by pitting, plowing and furrowing seen in Figure 9a–c. The average coefficient of friction after the fifteenth test cycle was 0.951.

## 4. Summary 

In this study, WC-Co cemented carbides were produced using a two-stage SPS with varying sizes of WC grain. The mechanical and tribological properties of these carbides were explored. Throughout the sintering process, Co, WC and Co_3_W_9_C_4_ were identified in all sintered variants. In particular, the areas with larger WC grains were apparent in the WC(0.1)µm_5Co composites having the smallest WC grains. The composites of WC (0.4) µm_4Co demonstrated the highest level of hardness, amounting to 2224 ± 19 HV30, which corresponded to the highest innate K_IC_ of 9.8 ± 0.4 MPa m^1/2^.

From the point of view of the tribological tests carried out on the produced sinters, it was observed that the effect on the improvement of the abrasive wear depended, among other things, on the average size of the manometric particles, the matrix content of cobalt and the parameters of the sintering process. The weight loss of the tested sinters did not exceed 1% of the initial weight after a distance of 2967 m. 

On the basis of the tribological studies, it was found that the optimal chemical composition was the WC (0.1μm)_5Co sinter, which was characterized by a mass loss that was lower by 75% and 205% compared with the WC (0.4μm)_4Co and WC (0.8μm)_4Co sinters, respectively. Only the classical abrasive wear phenomenon of the WC-Co sinters was observed. It was also found that no total wear of the sinters was observed for the test distance performed, and based on the course of mass loss kinetics, it was predicted that stabilized abrasive wear will occur at a distance of about 5000 m for the tested sinters.

On the basis of the obtained material test pits of the produced sinters, the authors concluded that the tested materials can be used for tools used in machining. In the next stage of the research, the authors will produce cutting blades from the sintered materials in order to analyze wear tests results under varying parameters of the turning process.

## Figures and Tables

**Table 1:** Cobalt powder composition.

Powder	Carbon wt%	Copper wt%	Iron wt%	Nickel wt%	Oxygen wt%	Silver wt%	Sulfur wt%
Cobalt	0.15	0.0050	0.020	0.30	0.60	0.0030	0.010

**Table 2:** Properties of the WC powder examined in this study.

Property	0.8 µm WC	0.4 µm WC	0.1 µm WC
Total C (wt%)	6.15	6.16	5.71
Free C (wt%)	0.03	<0.08	<0.05
Al (ppm)	<10	50	<10
Ca (ppm)	1	50	<10
Cr (ppm)	18	200	100
V (ppm)	-	280	<10
Fe (ppm)	86	200	200
Mo (ppm)	5	50	200
Si (ppm)	-	50	<10

**Table 3:** Types of WC-Co composites used.

Sample Designation	WC Initial Grain Size (µm)	Cobalt Content (% wt.)
WC(0.8µm)_4Co	0.8	4
WC(0.4µm)_4Co	0.4	4
WC(0.1µm)_5Co	0.1	5

**Table 4:** Hardnesses of the studied WC-Co samples.

Experimental Materials	Hardness, HV30											
	1	2	3	4	5	6	7	8	9	10	Mean Value	Standard Dev.
WC(0.8µm)_4Co	2027	1965	1973	1970	2026	2006	2006	1978	1944	1922	**2048**	**34**
WC(0.4µm)_4Co	2227	2205	2190	2226	2223	2256	2213	2220	2238	2238	**2224**	**19**
WC(0.1µm)_5Co	2148	2115	1994	2083	2094	2137	2102	2042	2042	2062	**2082**	**48**

**Table 5:** Average values of selected surface roughness parameters obtained after successive cycles.

Material	Cycle Number	Roughness Parameters	
		Ra (µm)	Rz (µm)
**WC(0.8μm)_4Co**	Initial state	2.54	7.70
	5	6.71	26.66
	10	7.49	27.84
	15	10.94	41.73
**WC(0.4μm)_4Co**	Initial state	2.31	10.74
	5	1.37	6.15
	10	1.77	8.11
	15	3.12	12.00
**WC(0.1μm)_5Co**	Initial state	3.01	12.30
	5	2.29	9.23
	10	2.12	8.49
	15	2.04	8.21

Ra—arithmetical mean roughness value; Rz—mean roughness depth.

## Data Availability

Data are contained within the article.
